# Critical incidents in anorexia nervosa: perspectives of those with a lived experience

**DOI:** 10.1186/s40337-021-00409-5

**Published:** 2021-04-19

**Authors:** Jenni Leppanen, Lara Tosunlar, Rachael Blackburn, Steven Williams, Kate Tchanturia, Felicity Sedgewick

**Affiliations:** 1 Department of Neuroimaging, Institute of Psychology, Psychiatry and Neuroscience, King’s College London, London, UK; 2 Department of Psychological Medicine, Institute of Psychology, Psychiatry and Neuroscience, King’s College London, 103 Denmark Hill, London, SE5 8AF UK; 3 South London and Maudsley NHS Foundation Trust National Eating Disorder Service, London, UK; 4 Psychology Department, Illia State University, Tbilisi, Georgia; 5 School of Education, University of Bristol, Bristol, UK

**Keywords:** Anorexia nervosa, Critical events, Positive experiences, Difficult experiences, Information processing bias, Thematic analysis

## Abstract

**Background:**

Although social-emotional difficulties are believed play a key role in anorexia nervosa (AN), there is uncertainty regarding what these difficulties might look like. Previous research has largely focused on a “disease model” of social-emotional processing in AN with little attention paid to positive emotions and experiences. Therefore, the aim of the present study was to obtain a fuller picture of critical life events as identified by those with lived AN experience.

**Methods:**

Thirty-four participants aged 16–48 with current or past AN completed an online survey describing self-defined positive and difficult critical events. Thematic analysis was used to assess patterns in participants narrative responses.

**Results:**

Two major themes were identified in the descriptions of positive critical events: *Moments of celebration* and *Unexpected positive outcomes*. These major themes revealed increased external focus and some corrective experiences that challenged the participants pre-existing expectations leading to new positive outcomes. Difficult events clustered into life events that were identified as *Eating disorder (ED) related* and *Non-ED related* and included the dimensions of relational conflict and feeling unsupported.

**Discussion:**

The findings suggest that although negative emotionality was identified in the accounts of those with lived experience of AN capacity for “*big-picture*” thinking with and explicit focus on others was also identified. Moreover, an openness to corrective experiences that worked to challenge negative expectations was evident for some participants. Together these findings have scope as targets for further clinical research and treatment interventions.

**Supplementary Information:**

The online version contains supplementary material available at 10.1186/s40337-021-00409-5.

## Plain English summary

Anorexia nervosa (AN) is a serious, life-threatening illness, and difficulties in emotional processing and social relationships are believed to perpetuate this illness by increasing isolation. However, what these difficulties might look like is uncertain. Furthermore, sole focus on such a “disease model” of AN has recently been challenged as it fails to consider positive emotions and experiences, and thus ignores the full human experience of someone with AN. This online study aimed to address this by asking people with lived experience of AN about both positive and difficult critical events. When describing positive critical events some participants discussed moments of celebration which reflected external focus and “big picture” thinking. Others discussed unexpected positive outcomes which revealed some negative biases that were met with positive actions. Difficult critical life events largely included discussion of relational conflict and loneliness, which for some participants took place against the backdrop of the eating disorder. These findings suggest that although AN may be largely characterised by negative emotions and loneliness, this is not the full picture. Many participants described living rich lives with both difficult and positive experiences and were open to have their negative expectations challenged with positive actions.

## Background

Anorexia nervosa (AN) is a complex eating disorder (ED) characterised by malnutrition and very low bodyweight [4]. Theoretical models of AN have suggested that social-emotional difficulties play an important role in perpetuating the illness [28, 73, 80]. Such difficulties are believed to lead to conflict in social relationships, which can in turn lead to increased isolation and create space for the ED to take over the person’s life [72, 73]. Although many interventions have been developed targeting various aspects of social-emotional functioning in AN [19], treatment response remains a significant challenge with only around 33% of patients reaching full recovery [1, 51, 66, 67]. Therefore, further investigation of social-emotional processing in AN could help improve understanding of the illness and aid the development of new interventions.

Over the years there has been a great deal of interest in examining social-emotional difficulties in AN using experimental paradigms. Large scale meta-analytic reviews of these studies report that people with AN have difficulties in several areas of social-emotional processing, including emotion recognition, theory of mind, and emotional expression [14, 59, 60, 65]. However, more recent work with larger sample sizes and utilising tasks with greater ecological validity, have found that people with AN have no general difficulties in explicit recognition of emotions or complex theory of mind tasks [2, 24, 82]. It has been suggested that previously reported difficulties in correct attribution of emotional states may be related to negative information processing biases in AN [3, 24], indicating that there is still a great deal of uncertainty regarding social-emotional difficulties in AN. Furthermore, over the past few years there have been calls to examine positive emotions and experiences in AN, highlighting the importance of studying resilience, hope, and optimism rather than focusing solely on difficulties [70]. At a time of uncertainty regarding the types of social-emotional difficulties that people with AN might have, it is valuable to gain further insight by adopting different methods and approaches.

As outlined above, the majority of previous work examining social-emotional processing in AN have been behavioural experiments or self-report questionnaire studies. Such quantitative work lacks the flexibility and ability to capture multiple aspects of behaviour and experiences that people may have. In contrast, a qualitative approach can provide unique insights into a variety of experiences and perspective on social relationships, which can further elucidate what social-emotional difficulties in AN might look like. The few qualitative studies in this area have documented that people with AN report difficulties with emotion expression, blocking or suppressing emotions, and in recognising their own emotions [49, 55]. Many also reported oversensitivity to, or misinterpretation of, others emotions, fixating on events or over-analysing the behaviour of others [46, 55, 79]. However, most of these studies have focused on exploring specific aspects of social-emotional processing, such as friendships or experience of emotions [79], or investigated patients’ views in the context of an interventional study [55]. A more implicit approach, examining broader emotional experiences of those with lived experience of AN with participants discussing key life events in their own life, expands the scope of qualitative studies on the topic of social and emotional processing.

To address the gaps in the literature outlined above, the aim of the present study was to shed light on both *positive* and *difficult* emotional experiences in AN by retrospectively exploring critical life events identified by those with a lived experience of AN in the acute phase of illness. We were specifically interested in events involving other people, as this would allow us to gain insight into social relationships and interactions during the acute stage of illness. Using the Critical Incident Technique (CIT [31];), we sought to answer the following research questions:

1. What are the important events participants choose to describe?
2. What emotional responses do participants report as linked to their recollections of positive and negative critical events?
3. What are the social relationships involved and how are they portrayed?

This study was exploratory in nature and therefore we did not seek to test a priori hypotheses.

## Method

### Participants

Thirty-four participants completed the study. All of the participants were 16 years old or older with current or history of AN. The sample demographic and clinical characteristics are presented in Table 1. Diagnosis was established by asking the participants to report if they had a diagnosis of AN and who diagnosed them (psychiatrist, psychologist, general practitioner, or other). All participants reported having been diagnosed with AN, ten of whom considered themselves to be recovered (REC). Twenty people reported having been diagnosed by a psychiatrist, 12 by their general practitioner, one by a clinical nurse specialist, and one by a clinical psychologist. As shown in Table 1, on average, the participants reported BMI below 18.5 but those who considered themselves recovered reported numerically higher than those with current AN. Additionally, on average our participants reported ED symptomatology, as measured by the Eating Disorder Examination Questionnaire (EDEQ), that was more than double of that reported by healthy individuals in the community [27]. Participants who reported being recovered reported numerically lower levels of ED symptomatology than those with current AN. Still, it is of importance to note that as this study was conducted online, no third person confirmation about the participants’ stage of illness or recovery was obtained. Twenty-three people also reported having other diagnoses in addition to AN, the most common of which were depression (*N* = 10, 43.48%), anxiety (*N* = 8, 34.78%) and obsessive compulsive disorder (*N* = 7, 30.43%). For further details please see Supplementary Table 1.

**Table 1:** Clinical and demographic sample characteristics

	Whole sample (*N* = 34)	Current AN (*N* = 24)	Recovered AN (N = 10)
Age M (SD)	26.43 (8.48)	27.11 (8.22)	24.81 (9.31)
Age at diagnosis M (SD)	16.97 (5.50)	17.88 (5.80)	14.80 (4.16)
BMI M (SD)	18.31 (3.13)	17.58 (3.17)	20.58 (1.05)
EDEQ Total M (SD)	3.25 (1.72)	3.93 (1.34)	1.48 (0.94)
Gender N (%)	Female 28 (82.4%) Male 4 (11.8%) non-binary 1 (2.9%)	Female 18 (75.0%) Male 4 (16.7%) Non-binary 1 (4.2%)	Female 10 (100%)
Marital status N (%)	Single 29 (85.3%) Domestic partnership 2 (5.9%) Married 1 (2.9%) Civil partnership 1 (2.9%) Separated 1 (2.9%)	Single 20 (83.3%) Domestic partnership 1 (4.2%) Married 1 (4.2%) Civil partnership 1 (4.2%) Separated 1 (4.2%)	Single 9 (90.0%) Domestic partnership 1 (10.0%)
Ethnicity N (%)	White 32 (94.1%) Mixed 1 (2.9%) Other/non-specified 1 (2.9%)	White 22 (91.7%) Mixed 1 (4.2%) Other/non-specified 1 (4.2%)	White 10 (100%)

*AN* anorexia nervosa, *BMI* body mass index, *EDEQ* eating disorders examination questionnaire, *M* mean, *SD* standard deviation, *N* number

The participants were recruited through online adverts on social media (Twitter, Facebook) and from ED charity (BEAT) website. As the study took place online, all the participants were required to complete an online consent form prior to completing the study. The study was approved by the King’s College London Psychiatry, Nursing and Midwifery Research Ethics Subcommittee, United Kingdom (HR-19/20–13,004). All procedures were conducted in accordance with the latest version of the Declaration of Helsinki (2013).

### Critical incident technique

Participants were asked to recount critical positive and difficult situations or events they had experienced while acutely ill using the CIT [31]. The CIT is an effective qualitative research technique used to systematically collect observations of significant events of which the person has first-hand experience. CIT has been used in numerous fields, including psychology, medicine, business, employee performance appraisal, and marketing [13, 22, 26, 34, 45, 64]. The CIT allows participants to self-define a critical or important event they wish to discuss further, rather than relying on the researchers’ views on what is considered critical. This approach empowers participants and actively includes them in the research process. As part of the CIT process, the participants are then asked variety of open-ended questions about the background of the event, the event itself, and the consequences of the event to gather as much information about the participants’ direct observations as possible. The use of such in-depth questions makes the CIT a particularly useful technique for research involving participants recalling important past events. Furthermore, the CIT technique places the focus of the analysis on the context of the event in addition to the event itself, enabling the researchers to explore the emotions, cognitions, and motivations surrounding it. Therefore, we used the CIT in the present study to enable us to reach our aims and answer our research questions.

The CIT is typically used as a semi-structured interview, but here it was adapted to questionnaire format (for original CIT and adapted version used in this study see Supplementary materials) to make the study more accessible and enable wider recruitment of participants. We created two CIT style questionnaires asking participants about critical positive and difficult life event. At the beginning of each questionnaire, participants were first asked to identify an event which included a social element, that is it involved some other people. Whether these other people were friends, family members, a partner, or someone else, was up to the participants. They were then asked for further details about the event as outlined above. Additionally, participants were asked how they felt about their own and the other people’s actions, whether they felt that the consequences were what they had hoped for, and how they thought the other people involved felt about the situation. All of the participants completed the CIT questionnaires.

### Qualitative data analysis

Participants’ responses to the CIT were subject to thematic analysis [9]. The thematic analysis was conducted by two authors, JL and FS. An inductive thematic analysis approach was chosen because it is primarily used to identify reoccurring similarities and differences in the narratives. In other words, this approach allowed data from multiple participants to be examined simultaneously, which in turn enabled us to meet the main aim of the present study. Additionally, inductive thematic analysis offers a flexible approach to data that enabled us to examine key positive and difficult social incidents in the lives of people with lived experience of AN and to explore other concepts such as different types of social-emotional difficulties, including information processing biases, emotion processing problems, and maladaptive emotion regulation styles.

Thematic analysis has been proposed to either involve inductive process whereby themes are identified from the data without prior expectations or theoretically driven analysis where the researcher’s prior knowledge of the phenomenon studied shapes the process [9, 10]. In the present study we used an inductive approach. CIT questions were used to probe participants to provide further detail about the event in question and the context surrounding it. Participants’ narrative responses to questions about positive and difficult events, were then analysed separately to identify commonly occurring themes and subthemes within each event category. We paid particular attention to: what were the social interactions that participants chose to discuss, how the participants described their own and others’ behaviour, what emotions were discussed or emerged from the narratives, and how satisfied the participants were with the outcome. Within each event category themes were identified without reference to previous work, thus representing inductive themes.

Before starting thematic analysis, all CIT responses were divided according to event category, descriptions of positive and difficult events were examined independently. We adopted a six-step approach to thematic analysis. We began with (1) data familiarisation, during which the narrative responses were read thoroughly. After this (2) initial codes were generated to identify content features, which represented commonalities and differences between the narratives. The feature codes were then grouped and (3) broader themes were identified. Following standard practice, during this stage, certain content feature codes were discarded if they were identified in fewer than 3 participants’ narratives or if they did not fit in any of the other broader themes already generated. Next the themes generated by grouping the feature codes were (4) reviewed to ensure clear relationship between themes and subthemes and avoid duplication. After review, (5) themes and subthemes were defined according to the concepts they represented and the (6) final report was produced. The codes and themes were agreed upon independently by the two authors. Any disagreement or uncertainty regarding the codes and themes were brought to the whole team for further discussion.

## Results

### Positive critical events

**Fig. 1:**
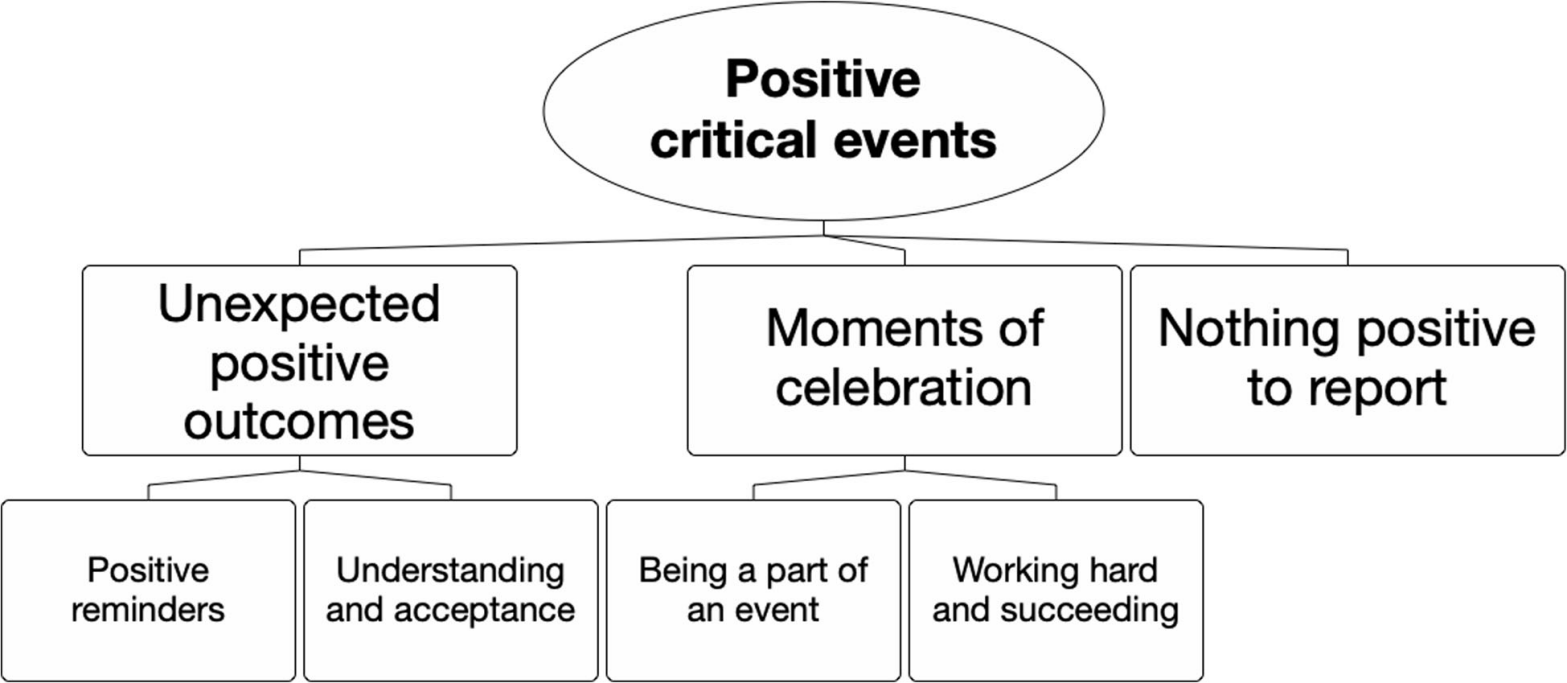
Positive critical events. The map shows themes that were identified in participants’ narrative descriptions of positive important events

When the participants were asked to reflect on positive critical events, some discussed events that were positive throughout and left them feeling good about themselves. However, most participants chose to discuss events that had at least some negative components, either reflecting their own negatively biased expectations or focusing on negative events in a perceived absence of positive experiences. Thus, three major themes reflecting different emotional valences were identified in participants’ narrative responses to the CIT questions about positive critical events: (1) Unexpected positive outcomes, (2) Moments of celebration, (3) Nothing positive to report (Fig. 1). Each theme and their corresponding subthemes are presented in Fig. 1 and discussed further below.

#### Unexpected positive outcomes

The *Unexpected positive outcomes* theme was identified in several narrative descriptions of events where the participants described feeling low and alluded to having negative expectations of themselves, the event itself, or the other people involved. Instead of having these expectations met, the participants talked about how they were pleasantly surprised by the actions of others: *“My sister found my food that I was hiding behind my bed. It was positive because [she] wasn’t rude and horrible about it” (AN-05)*. This theme suggests that although many participants recognised having negative information processing biases, assuming they were worthless and unloved, or the people around them did not care or understand them, they were open to having these biases challenged. Despite such negative context surrounding the events, it was clear from participants’ descriptions of the outcomes that these were critical positive experiences.

The *Unexpected positive outcomes* theme comprised of two subthemes: (a) *positive reminders* and (b) *understanding and acceptance*. Several participants discussed receiving *positive reminders* from their loved ones during times when they were feeling low or going through hard times. In all these narratives, our participants briefly alluded to their low self-esteem and low mood, which they assumed their loved ones knew about. This knowledge was discussed as the catalyst that led their loved ones to show the participants through actions that they were indeed loved and worthy. *“My chest infection had been worsening in the few days preceding this incident, and in turn my mood had dropped quite a bit, so my self-esteem had been particularly low...Two of the day staff volunteered to take me [to the Emergency department] … them going above and beyond made me feel cared for and worthy” (AN-07)*. A few participants also mentioned that although the positive reminders made them feel loved and gave them motivation to work hard on their recovery, they also triggered feelings of guilt: *“I was in hospital on Christmas day and my father visited. He brought with him a big jar of notes that my friends had written as well as small gifts from them, all [the] memories from the past and things they liked about me … I felt very emotional, but also guilty” (REC-05)*. This further indicates that some people with AN may battle with a deep sense of worthlessness such that even positive exchanges can give rise to negative emotions, which in turn can make it difficult to cope with or accept these positive interactions. This illustrates the complexity of emotional experiences among people with AN.

Another subtheme was *understanding and acceptance*, which included discussion of significant stages of illness. These included having other people find out about ED related behaviours and needing hospital treatment. Hiding the illness is common in AN, often fuelled by feelings of embarrassment, shame, or desire to protect the illness, which can be seen as something valuable and positive. This, in turn, often leads to avoidance and further social isolation, creating barriers to help-seeking. Although all the participants talked about how other people discovering their ED was a daunting prospect, they also discussed their desire for support and acceptance: *“[I] wanted no one to find out. But it was good that they found out … I felt supported and less alone with what I was doing” (AN-05)*. Being met with compassion and understanding also served as an opportunity for more open discussion and learning in the context of treatment: “*I have a tendency in these [confusing and upsetting] situations to withdraw from conversations and this was noticed by the therapist … Our sessions individually had initially focused on her gaining an understanding of my personality and thought patterns … “[It] planted the seed around navigating through negative emotions, rather than around them, and the skills I developed during this time enabled me to communicate this better to the people around me” (REC-02).* It was clear in the narratives that understanding and acceptance from loved ones and professionals was instrumental to enable the participants to communicate without judgement, and direct compassion towards themselves and allow themselves to engage with treatment.

#### Moments of celebration

The theme *Moments of celebration* included written accounts of being part of a fun event, such as a wedding or a birthday celebration, and achieving goals, such as getting accepted into university. The participants described the events using language that suggested they felt the events were positive throughout. Interestingly, this theme was generally characterised by an external focus of attention, with the participants primarily describing the event and the other people involved. This could reflect an attempt to reduce self-focused attention to lower social anxiety. Indeed, a few participants discussed how focusing on activities and their loved ones enabled them to see the “*big picture*”, *“get out of [their] own head” (REC-01)* and enjoy the moment. This theme comprised of two subthemes, (a) *Being a part of an event* and (b) *Working hard and succeeding*, which are discussed further below.

The first subtheme, *Being part of an event*, included recollections of both significant occasions such as a wedding or birth of a relative, as well as smaller get-togethers, including birthday celebrations and days out. All narratives within this subtheme were characterised by reduced self-focused attention and active involvement, and the participants discussed how being part of the event and focusing on the activities made them feel more hopeful and connected to their loved ones: *“By saying ‘yes’ to one thing, and seeing I was still okay after it, I was more likely to say ‘yes’ to the next. As I engaged in more things, I enjoyed myself more as had more confidence that the consequences would be okay.” (REC-07).* It was clear this sense of belonging and shared experiences made participants feel like they were able to function competently as part of their community, which for some participants served as motivation to strive towards recovery form AN: *“His [nephew’s] birth gave me the determination to work hard on my recovery so that I could watch him grow into a bright young boy and spend valuable time with him” (AN-08)*.

Another subtheme was *Working hard and succeeding* which was characterised by the participants discussing how they worked hard to reach their goals. Although some participants mentioned how receiving support and encouragement from their loved ones during this time was important in keeping them focused and calm, the primary focus was on their own work and efforts: *“I worked hard to get where I wanted to go [University of choice] … [my family offered] Lots of support and encouragement which helped” (AN-14).* This sense of accomplishment made people feel proud and competent: *“Having been so unwell that I had previously been unable to undertake barely any physical activity … [I] felt a huge sense of achievement in being able to take my young daughter onto the ice rink” (AN-09)*. It was clear that the feeling of achievement allowed individuals to view themselves in a more positive light and feel more capable, which in turned strengthened their self-compassion and sense of self-acceptance.

#### Nothing positive to report

Unexpectedly, three people recounted negative experiences with no clear positive themes as their positive critical events. These included accidental death of a loved one and being victimised. One person also talked about missing out on an otherwise important, positive family event: “*My brother asked his girlfriend to marry him … I missed him asking her … I felt like a failure and a let down to the family … [The others] never spoke to me about it. They just left me. It was just my brother that said I’d upset him by not coming.” (AN-09).* Although it was clear from the quote that celebrating and being part of this important event would have aligned with their values, the narrative description of the memory was very different compared to those in 5.1.2. Their focus was largely on letting others down and feeling guilty about doing so, rather than on celebrating a positive experience with loved ones. A tendency to associate the self with negative experiences is common in AN [61], and these narratives could be a reflection of an extreme case of negative bias whereby these participants felt that they had nothing positive to discuss or that nothing positive had happened to them. Alternatively, these narratives may reflect the number of significant negative life events our participants have experienced. Indeed, people with AN have been found to report more negative and stressful experiences than a healthy comparison group [20], as well as other psychiatric inpatients including people with depression and anxiety [44, 76].

### Difficult critical events

**Fig. 2:**
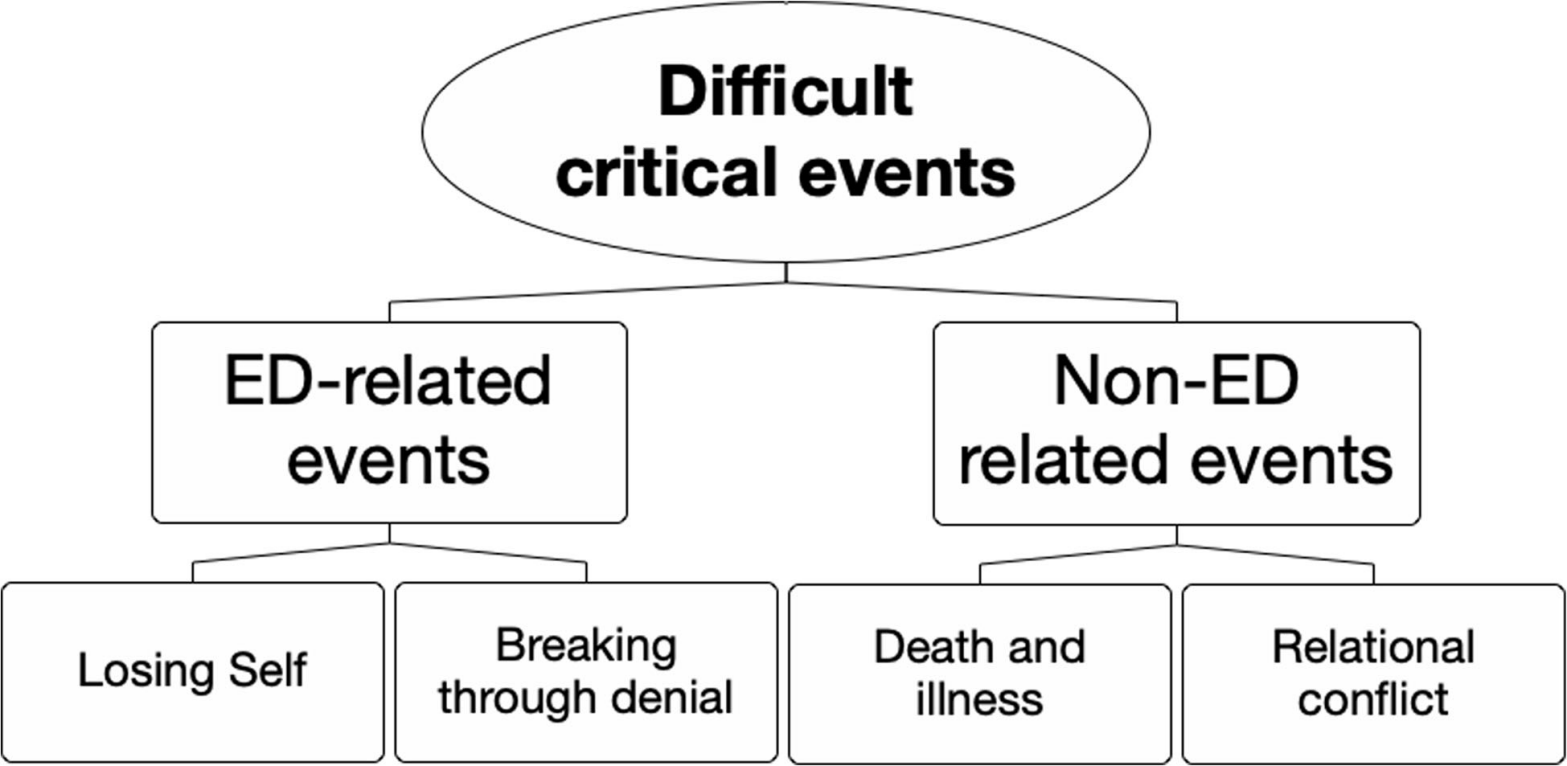
Difficult critical events. The map shows themes that were identified in participants’ narrative descriptions of difficult important events. ED = eating disorder

When asked to recount difficult critical events in their lives, twenty participants discussed situations that were in some way related to their ED. It became clear that for these individuals, the AN was often at the centre of important negative social experiences. This is to be expected as the illness and associated symptoms often serve as both the cause and consequence of shame and distress. Interestingly, the other 14 participants discussed difficult events that were not necessarily directly tied to AN, but were to do with other distressing life events that happened to occur when they were ill. Thus, the following themes were identified in the narratives: (1) ED-related difficult events and (2) non-ED difficult events. The themes and their corresponding subthemes are presented in Fig. 2 and discussed further below.

#### ED-related difficult events

The *ED-related difficult events* theme was identified in narratives where people wrote about interactions with their loved ones or ED services that revolved around the illness and which resulted in conflict and personal distress. The participants primarily wrote how other people’s reactions to the illness or associated behaviours made them feel pressured or ashamed. This theme suggests that people with AN have many complex emotions tied to their illness, which can be difficult to navigate during stressful events, causing them to react in ways contrary to their values of belonging and care for the people around them. Indeed, people wrote how these difficult interactions left them feeling alone and unsupported, but also how the illness made them feel like the odd one out and isolated them from their loved ones. It was clear that these social events formed a significant step in their illness journey. The *ED-related difficult events* theme comprised of two subthemes: (a) *Losing self* and (b) *Breaking through denial,* all of which are discussed in more detail below.

As part of the first subtheme, *Losing self*, people wrote about a particularly turbulent time in their lives when AN was dominant, disrupted their daily lives, and prevented them from engaging with activities in line with their values and desires. Several participants implied that they felt unable to stop themselves from engaging in ED behaviours and cognitions, which frequently led to personal distress and relational conflict. Social eating especially made participants feel pressured to challenge their ED thinking style and behaviours, which they felt unable to do, resulting in negative outcomes: *“Family dinner and [I] had to choose what to eat and how much [I] wanted from each part. [It was] difficult because it was hard to know if eating too much or too little and difficult because my family saw me hide it in my sleeves and it was embarrassing … my sister shouted at me” (AN-05)*. Individuals also wrote about how they wanted to join in and be part of activities but the illness made them feel it all impossible, *“Went out for dinner and ended up crying because I ate” (AN-01)*, which led to further feelings of guilt and shame: *“I upset my family and ruined the evening. Also embarrassed myself … I didn’t want to react that way but at the time it helped me cope.” (REC-01)*. These recollections convey a feeling that losing oneself to AN is very isolating experience.

Other participants found that being under pressure frequently resulted in conflict and made them feel misunderstood or ignored when they tried to explain or express their feelings and thoughts. In these narratives people wrote about engaging in behaviours and thinking styles that could be interpreted as protecting the illness identity. Indeed, several participants talked about relational conflict in a manner that suggested a *me-*versus*-them* mentality: *“As my mum and I exited the ward that Friday night, my care-coordinator handed a meal plan for that weekend to my mum. It wasn’t the one we had discussed … My anorexia was talking for half of the conversation, trying to get my mum to change the meal plan … I felt extremely betrayed both by my care-coordinator (who I got on with and thought I could trust) and my mum (who I felt was taking the hospital’s side and not her own daughter’s)” (REC-07)*. Frequently, people also reported that these difficult interactions solidified their impressions that their loved ones were not there to support them when they were overwhelmed by the illness, so could not reach out and ask for help when they needed it: *“I felt belittled, like she [a friend] didn’t really care. I felt scared that others felt the same as her, and that I shouldn’t talk to others about how I felt because they would start resenting me” (AN-19)*. Interestingly, in all these narratives, the participants conveyed a feeling that things could have turned out better if they had been heard instead of being dismissed and rejected.

*Breaking through denial* was another clear subtheme, including discussions of situations where individuals were suddenly made aware of the extent of their illness and how it impacted other people around them. Denial of the illness or its impact on the person’s health is common in AN. This thinking style can be thought of as a coping mechanism, which can help the person feel strong and in control whilst simultaneously becoming increasingly isolated and being at the mercy of AN. Thus, unsurprisingly some participants seemed shocked to receive diagnosis of AN when they were going to see their doctor for other reasons: *“I was diagnosed with anorexia nervosa and was about to be hospitalized … I did not believe this was happening. I did not think there is anything wrong with me. I have heard about anorexia, but I did not think I am the one having it.” (REC-10)*. These participants also alluded to often being unaware of the impact AN had on their loved ones, which made having to suddenly face the reality of the illness an even more difficult experience: *“I passed out at the GP and it made my mum very upset … I did not want to upset or worry my mum.” (REC-03)*. In this recollection the participant was concerned about how she was consumed by the illness to the extent that it made her blind to the impact it was having on not only on herself but her loved one. In the end, despite the difficulties, some participants also wrote about positive outcomes and relief associated with having to face their illness: *“I was almost relieved to be diagnosed, it felt like I could finally speak to somebody about it.” (AN-10)*.

#### Non-ED related difficult events

The narratives that formed the *Non-ED related difficult events* theme involved negative life experiences that were not directly related to the ED, but rather happened to occur while the participants were ill. These primarily included recollections of very difficult experiences, which the participants struggled to cope often resulting in feelings of loneliness and shame. Other people’s and their own reactions often compounded these feelings later on, further deepening their experience of social isolation. This theme comprised of two subthemes: (a) *Death and illness*, and (b) *Relational conflict*.

Under the *Death and illness* subtheme, our participants discussed grieving the loss of a loved one or dealing with a family members illness. When discussing losing a loved one, the participants described going through common stages of grief, but appeared to react in one of two ways: some felt upset and overwhelmed to the point that they either did not want, or know how, to reach out for help: *“I was on the way to a party on the bus and got a message from a friend that our friend had died. I continued to the party and didn’t mention it to anyone there … I felt very dissociated and didn’t know what to do. I got quite upset and no one knew why. I felt very alone and distressed when I should’ve gone home and spoken to someone about it.” (REC-05).* Other participants, on the other hand, talked about how the loss made them feel exasperated: *“The death of my godmother/aunt. I was feeling very angry as she was sick and I wanted to swap places with her” (AN-16)*. A few participants talked about how their need to stick to their routine at the time of the incident made them feel guilty and ashamed later: *“My mum had a seizure … I was upset and frustrated as I stressed about how I’d get to work and who would make my lunch. I should’ve been more upset and caring and stayed at home” (AN-04)*. This could reflect cognitive rigidity and resistance to change common in AN, which can prevent a person from behaving and responding in accordance with their value of compassion towards others and themselves causing further distress and shame.

The other subtheme identified in the narratives was *Relational conflict*. This subtheme included discussions of difficult interactions with loved ones which left participants feeling alone and upset, when what they desired was support and encouragement from their loved ones: *“I was in hospital after having a blackout and badly injuring my face, and I texted [my friend] to let her know, but received an abusive reply … She told me our friendship was over” (AN-18)*. Interestingly, similar to ED-related relational conflict discussed above, the narratives under this subtheme also included discussions of how these difficult interactions left the participants feeling dismissed, which was often interpreted as an indicator that nobody truly cared and there would be no point in reaching out to others in the future: *“My mum disregarding my feelings made me feel invalidated and added to my belief that I shouldn’t open up to people.” (AN-22)*. Taken together, this could be one of the factors influencing the development or maintenance of negative cognitive biases in AN.

## Discussion

The present study aimed to explore positive and difficult events in the lives of people with lived experience of AN. The participants completed two CIT-style questionnaires asking them questions about these critical events and the resulting narratives were subject to thematic analysis. Many of the themes identified in the positive and difficult narratives were presented against the backdrop of the eating disorder, which formed a large part of the participants’ lives when they were acutely unwell. Importantly, the findings suggest that individuals with lived experience of AN have the capacity, even in the acute stage of the illness, to engage and recount rich, enjoyable experiences demonstrating optimism and hope.

Over the past few years there have been calls to study positive emotions in AN [70], however, to our knowledge no studies have thus far explored narratives of positive experiences among this group. In the present study, the critical events that were entirely positive under the theme *Moments of celebration* frequently involved discussions of achievements, having something to look forward to, and active engagement in activities. Such focus on other people and future events could reflect reduced self-focused attention. Self-focused attention, sometimes referred to as rumination or dwelling on one’s own emotions and internal experiences, has been extensively studied in the field of social anxiety. Reduced self-focused attention been associated with less negative interpretation bias and reduced tendency to exaggerate others’ negative feelings towards oneself [5, 8, 56]. Reducing self-focused attention, particularly to negative aspects of the self, has been found beneficial in reducing social anxiety and fear of negative evaluation [8, 25, 43, 48]. This finding suggests that people with AN can shift their focus of attention and have rich, enjoyable experiences, demonstrating optimism and hope even in the acute stage of illness.

Fully engaging in activities and focusing on other people can also be interpreted as increased *“big picture”* oriented thinking and living authentically in accordance with their personal values. Experimental research within the general population has documented that increased *“big picture”* thinking is associated with a tendency to react in a positive manner and increases positive affect [33, 42]. Interventions targeting excessive detail focus in AN, such as cognitive remediation therapy, have been found to improve mood and increase “*big picture*” thinking in experimental tasks [23, 37, 50]. Similarly, authenticity and openly living according to one’s own values has been associated with increased positive affect, while inauthenticity has been reported to be associated with negative affect, increased rumination, and lack of clarity about one’s own emotions [41, 54]. Furthermore, qualitative research has found that the ability to see the *“big picture”,* and living according to own values of care, may be a key component in supporting recovery from AN [15, 18, 78]. When discussing their recovery journey, people with lived experience of AN have reported that focusing more on their loved ones enabled them to start to questioning the illness and recognise its full impact on their own and others’ lives [78]. This was identified as a turning point in the illness journey at which the person felt able to start distancing themselves from the illness and to develop an identity independent of AN. Taken together, these findings suggest that further investigating and building on the strengths and resilience people with AN have may be a useful target for further interventions. Sole focus on a “disease model” of emotional processing in AN often fails to examine the full human experience of someone with AN [70].

In the present study, several participants seemed to demonstrate negative biases when asked to discuss important positive events. This is in line with previous experimental work finding that people with AN show increased attention towards negative stimuli and a tendency to interpret ambiguous scenes in a negative way [3, 16, 17, 74]. Interestingly, our participants found these experiences to be positive because their own negative expectations were met with positive actions from the people around them, particularly when they were met with support, validation, and understanding that they may have felt unable to ask for. These events could be interpreted as so-called corrective experiences, in which a person challenges their own biases and expectations resulting in a new, often more positive way of viewing others and the self [38]. Corrective experiences are argued to play a key role in the transformative processes promoted in psychotherapy and are associate with breakthroughs in a person’s willingness to feel previously demonised and feared emotions and adopt new behaviours [21]. Corrective experiences have been reported to provide people with new understanding of previous important experiences through reflection [35, 36, 81]. This in turn leads to improved self-esteem, reduced reliance on defence mechanisms, and greater social support achieved through disclosure and sharing of emotional experiences [35, 36]. Although corrective experiences have not been extensively researched in the field of ED, one study examining the usefulness of psychodrama reported that by creating corrective experiences, the group allowed the participants with ED to re-evaluate their previous emotional, personal, and cognitive experiences [63].

In previous qualitative work, people with AN highlighted the importance of allowing themselves to accept social support and open up to others when recounting their experiences of recovery [29, 47, 78]. These participants engaged in careful expectation management, first gauging others reactions before opening up, which in the end gave them the affiliate support they were seeking, aiding their recovery [29, 78]. Taken together with the present findings, this suggests that people with AN are open to corrective experiences, which highlights need for further research into how these experiences may be facilitated in a therapeutic context. Importantly in the present study, a few participants discussed how the positive actions and gifts from others made them feel loved, but also triggered feelings of guilt. This could be interpreted as evidence of an internalised sense of worthlessness and shame, which makes believing and accepting positive words and actions towards oneself difficult. Indeed, previous work has found that people with AN frequently report feeling worthless, which can interfere with social relationships and recovery [29, 58, 71, 77]. Taken together, these findings suggest that negative cognitive biases can be challenged with corrective experiences in AN, but the actions and reactions of others, and navigating through feelings of worthlessness are key in order to not further alienate the person.

Difficult critical events could be broadly divided into ED-related and non-ED events. When the participants were discussing ED-related difficult events they talked about how losing self to the illness led to personal distress and relational conflict, and how breaking through denial forced to face the consequences of the illness. Our participants mostly talked about how the illness made them feel like the odd one out and how others’ behaviours and reactions made them feel pressured, misunderstood, and alone, with some participants talking about how they wanted to escape situations where they felt unable to cope. Previous studies with people with history of AN have reported that feeling alone with the illness and unheard or misunderstood made participants feel less safe and more reluctant to accept changes [29, 32, 52, 53]. This led to increased distrust between the patient and their loved ones, enforcing feelings of loneliness, and negatively impacted the therapeutic alliance [32, 52]. As a result, participants reported that they often fell intentionally silent and did not want to reach out. However, such reduced expressiveness and responsiveness has been found to be associated with reduction in social support offered by others and disrupted communication [11, 12, 40]. Combined, this may create a vicious cycle of reduced social support and isolation, leaving the person at the mercy of the illness.

Interestingly, a few participants who discussed breaking through denial as a critical difficult experience also found that in the end these were key events in their recovery. This could be taken as *“hitting rock bottom”* and suggests that having to finally face the impact the illness had on them and their loved ones was an important source of motivation for these participants. Together with the experience of losing self to the illness, these events enabled the participants to witness how the illness prevented them from living in accordance with their personal values. Indeed, many participants wrote about how they cared for their loved ones and didn’t want to upset them, but they felt they weren’t strong enough to fight or challenge the illness, which then led to the conflict. This could be taken as a shift in thinking, moving from denial to acknowledging the impact of the illness. Indeed, previous studies have reported that people who have recovered from AN found having to face the personal, health, and social consequences of the illness was a turning point in their illness journey [47, 78]. It enabled them to see the *“bigger picture”* and how their actions impacted others around them, which in turn gave them motivation to work on the their recovery [78]. These findings suggest that by reflecting on difficult life events some people were able to turn them into a positive creating more space for and motivation for change. These findings highlight the importance of examining how AN impacts an individual’s ability to live authentically in accordance with their values, which in turn could be an important target for interventions helping the individual distance themselves from AN.

When recounting both ED-related and non-ED difficult life events several participants wrote about relational conflicts as particularly important interactions. Our participants wrote about how others’ negative reactions to their attempts to seek support or ED-related coping strategies made them feel alone and rejected, which were in direct contrast with the understanding and supportive reactions described under the theme *Unexpected positive outcomes*. Many participants also mentioned how these interactions served to further enforce their pre-existing notion that other people did not truly care about them and that they were alone with their illness. Such feelings of loneliness and rejection, possibly compounded by past traumatic and painful experiences, could be one of the factors influencing the development and maintenance of negative cognitive biases in AN, similar to those alluded to in the *Unexpected positive outcomes* theme. This could be interpreted as the formation of a maladaptive coping strategy founded in negatively valenced expectation management, whereby the person begins to expect others to respond in a negative fashion based on previous experiences. A tendency to generalise others’ negative feelings towards oneself has been associated with increased negative self-focused attention, poor social functioning, and increased rejection sensitivity [5, 6, 8, 56]. Previous experimental studies have reported that people with AN report elevated rejection sensitivity, perceived lower social rank, and reduced social adjustment [15, 18, 62, 69, 75]. When expecting to be rejected by others, people who experience high rejection sensitivity may engage in passive, hostile behaviours including avoidance and withdrawal of love and support [6, 7]. Such strategies then tend to become self-fulfilling prophecies, often leading to more social difficulties, including further social rejection [6, 7]. However, as shown in the present study, people with AN are open to having these maladaptive cognitive biases challenged through positive actions. Further investigation of the development of negative cognitive biases in AN, their role in illness maintenance, and strategies for challenging them, may be of interest.

### Research and clinical implications

The present study highlights the importance of studying both positive and negative life experiences in AN which may form the foundation of the behaviours documented in previous experimental work. Future research could benefit from investigating how difficult social interactions and relational conflict shape the perceptions of people with AN and how these interactions may influence illness progression. The present findings suggest that participants’ negative interpersonal experiences may influence the development of cognitive biases and possible maladaptive coping strategies. Maladaptive coping styles, including experiential avoidance and unwillingness to share own experiences, have been found to be associated greater illness severity and worse outcome in AN [57, 73]. This finding is relevant for clinical work highlighting the importance of focusing on relationships and trauma in the treatment of individuals with AN. Interventions, such as family therapy, aim to target some of these factors, namely family dynamics, and there is some evidence that family therapy can be effective in promoting remission [30]. It would be of interest to build on these findings and examine the effectiveness of interventions that target social relationships and complex trauma more broadly, reaching outside of the immediate family.

Importantly, the present findings demonstrate that people with lived experience of AN recount rich, enjoyable social experiences, as well as showing optimism and hope, even in the acute stages of illness. These findings highlight that people with AN can and do demonstrate strength and resilience and future research may benefit from further exploring the role of these positive traits in AN. Such an approach would not only allow us to explore the full human experience of someone with AN [70], but also help capitalise on strengths, such as “*big picture*” thinking, which has been found to play a key role in recovery [78], when developing new interventions. Shifting focus from the “disease model” of AN towards building on strengths can help support existing clinical interventions, increase motivation, and help the individual feel more confident in being able to reach recovery.

In the present study many participants alluded to the role of personal values in their recollections of positive and difficult life events. Positive life events were associated with feeling able to live authentically, in accordance with their value system. Difficult life events, on the other hand, especially those related to the illness, were associated with shame and distress caused by failing to live up to one’s own values, such as value of caring for others. These findings highlight the importance of knowing oneself and one’s identity outside of AN in the recovery journey. Maintaining the illness can contradict things an individual values about themselves, such as caring for family members but knowing that you are upsetting them. Recognising this tension could help an individual to distance themselves from the illness creating opportunities for change and aiding recovery. To our knowledge, previous work exploring personal values in AN have largely focused on examining illness-related values such as the value of thinness [39, 68]. Therefore, further research into personal values that extend beyond AN and investigation of interventions that capitalise on strengths and values outside of the illness would be of interest.

### Limitations

The main limitation of the present study was that it was conducted online. This may have limited the extent to which participants felt able to disclose painful negative events and how they interpreted them. Additionally, as the study was conducted online, we were not able to obtain third person confirmation of participants stage of illness or recovery. This limits the conclusions that can be drawn from this study. Future studies are needed to confirm the present findings using methods that make participants feel safe to disclose and discuss difficult life events. Additionally, conducting the study online meant that it was not possible to conduct a full assessment to confirm diagnosis or stage of illness. Additionally, the sample was almost entirely white, British, and female, which limits generalisability. Future studies may benefit from targeting specifically gender- and ethnic minorities as their experiences of AN and related difficulties are likely to be different.

The CIT was adapted into questionnaire format, which allowed us to recruit participants from more widely across the country without placing an additional burden on participants. However, this format limited the responsiveness inherent to an interview setting. For instance, had the study been conducted face-to-face, it would have been possible to ask why participants described negative experiences when asked for positive events. Since only a few participants recounted negative or upsetting memories when asked about positive critical events, it is possible that these participants misunderstood the question.

## Conclusions

The present study aimed to investigate positive and difficult critical incidents in AN. Several participants talked about positive events that involved *Moments of celebration*, with focus on reduced self-focused attention, ability to live authentically, and increased *“big-picture”* thinking. Still, the majority of the positive critical events also involved some negative experiences that then ended on a positive note. When discussing these *Unexpected positive outcomes* participants revealed some evidence of openness to corrective experiences to challenge their negative biases. Difficult critical events broadly involved discussion of *ED-related* and *Non-ED life events*. When discussing *ED-related life events* participants wrote about how losing themselves to the illness made them feel like they couldn’t join in even when they wanted to and prevented them from behaving in accordance with their personal value system. Some participants also recounted how others around them made them feel under pressure, asking for too much too soon ultimately resulting in relational conflict. Interestingly, relational conflict appeared in both ED-related and Non-ED related life events and discussion of how these conflicts solidified participants pre-existing expectations that other did not truly care about them and convinced them. These findings suggest that negative experiences may play a role in the development of negatives biases and possible maladaptive coping strategies in AN. Importantly, however, it seems that these negative biases could be challenged through corrective experiences, suggesting that it is possible to modify negative biases in AN. Moreover, the present findings highlight the need for further research into personal values in AN that extend beyond the illness such as value of caring for their loved ones.

## Supplementary Information

**Additional file 1.**

**Additional file 2.**

**Additional file 3.**

## Data Availability

The CIT questionnaire used in the study is included in this published article (Supplementary materials). The datasets generated during and/or analysed during the current study are not publicly available due to the data containing information that could compromise research participants’ privacy/consent, but are available from the corresponding author on reasonable request.

## Abbreviations

AN: Anorexia nervosa
ED: Eating disorder
REC: Recovered
CIT: Critical incident technique
BMI: Body mass index
N: Number
M: Mean
SD: Standard deviation
