# Supplementary material for: Critical incidents in anorexia nervosa: perspectives of those with a lived experience
Source: J Eat Disord. 2021 Apr 19;9:53. doi: 10.1186/s40337-021-00409-5 (PMC8054426; doi:10.1186/s40337-021-00409-5)
Supplement: Supplementary file 1 — Additional file 1. [file 40337_2021_409_MOESM1_ESM.docx]

Supplementary Table 1. Other diagnoses participants reported having

| Diagnosis | N | % |
| --- | --- | --- |
| Depression | 10 | 43.48% |
| Anxiety | 8 | 34.78% |
| Obsessive compulsive disorder | 7 | 30.43% |
| Generalised anxiety disorder | 6 | 26.09% |
| Autism | 2 | 8.70% |
| Eating disorder not otherwise specified | 2 | 8.70% |
| Epilepsy | 1 | 4.35% |
| Emotionally unstable personality disorder | 1 | 4.35% |
| Borderline personality disorder | 1 | 4.35% |
| Attention deficit hyperactivity disorder | 1 | 4.35% |
| Binge eating disorder | 1 | 4.35% |
| Obsessive compulsive personality disorder | 1 | 4.35% |
| Body dysmorphic disorder | 1 | 4.35% |
| Dermatillomania | 1 | 4.35% |
| Ulcerative colitis | 1 | 4.35% |
| Rheumatoid arthritis | 1 | 4.35% |
| Irritable bowel syndrome | 1 | 4.35% |
