# Supplementary material for: Critical incidents in anorexia nervosa: perspectives of those with a lived experience
Source: J Eat Disord. 2021 Apr 19;9:53. doi: 10.1186/s40337-021-00409-5 (PMC8054426; doi:10.1186/s40337-021-00409-5)
Supplement: Supplementary file 3 — Additional file 3. [file 40337_2021_409_MOESM3_ESM.docx]

Interviewer sample of a form for the use of the Critical Incident Technique (Flanagan, J. C. (1954). The critical incident technique. Psychological bulletin, 51(4), 327.)


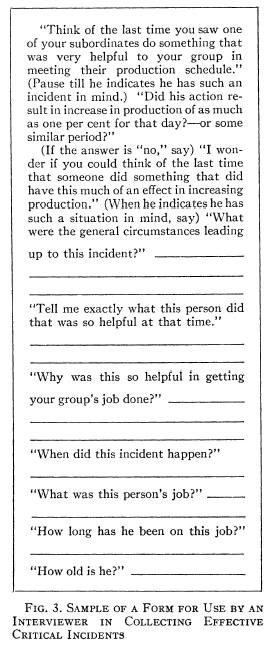


CRITICAL INCIDENT QUESTIONNAIRE (version 1, 29/04/2019)

In the following questionnaire you will be asked to cast your mind back to some critical, significant moments in your life while you were acutely ill. By incident we mean any activity that involved you and other people who you are close to, including family members, friends, or other loved ones. Critical incidents are any incidents that had particular importance to you and that had a substantial impact – positive or negative – on the activity or on your day.

As a part of this questionnaire, you will be asked to tell stories about positive, surprising, and difficult incidents that have happened to you. These stories will be followed by further questions about the details surrounding, that led to, and followed the incident. For these reasons, please think carefully what type of incident you feel comfortable with disclosing and discussing in detail.

If discussing these incidents are making you feel upset, distressed, or anxious, please take a break. You can always close the survey and come back to it at a later date using your login details.

If you need further support please contact you care team or GP. We have also listed below a few websites that provide online support:

<https://www.beateatingdisorders.org.uk/>

<https://www.mind.org.uk/>

<https://www.rethink.org/>

<https://www.samaritans.org/>

You can also call the Samaritans crisis line free of charge if you need support:

**116 123**

You can also call the NHS 111 if you need urgent, non-emergency support.

If you feel that you are in a mental health emergency and need urgent help please go to your nearest A&E or call 999 immediately.

**POSITIVE CRITICAL INCIDENT**

Please take 10 minutes to think about a **positive** critical event that occurred while you were acutely ill and involved you loved ones or other people you are/were close to. Please choose the event carefully as we will be asking you further questions in the next few pages.

Please answer all the questions in your own words.

1. Please tell us about the incident or event. What happened? What was particularly positive about it?

|  |
| --- |

1. What do you think led to or preceded it?

|  |
| --- |

1. About you:
   1. What were you doing during the incident or event?

|  |
| --- |

- 1. What was the result of your actions?

|  |
| --- |

- 1. Was this the result you wanted / had hoped for?

|  |
| --- |

- 1. Why were these actions effective or ineffective with regards to your desired outcome?

|  |
| --- |

1. About the others who were involved:
   1. What were the other people doing during the incident or event?

|  |
| --- |

- 1. What was the result of their actions?

|  |
| --- |

- 1. Do you think this result was what they were hoping for?

|  |
| --- |

- 1. Why were these actions particularly effective or ineffective with regards to the result of their actions?

|  |
| --- |

1. What would you do if you found yourself in the same situation today?

|  |
| --- |

If discussing these incidents are making you feel upset, distressed, or anxious, please take a break. You can always close the survey and come back to it at a later date using your login details.

If you need further support please contact you care team or GP. We have also listed below a few websites that provide online support:

<https://www.beateatingdisorders.org.uk/>

<https://www.mind.org.uk/>

<https://www.rethink.org/>

<https://www.samaritans.org/>

You can also call the Samaritans crisis line free of charge if you need support:

**116 123**

You can also call the NHS 111 if you need urgent, non-emergency support.

If you feel that you are in a mental health emergency and need urgent help please go to your nearest A&E or call 999 immediately.

**DIFFICULT CRITICAL INCIDENT**

Please take 10 minutes to think about a **difficult** critical incident that occurred while you were acutely ill and involved you loved ones or other people you are/were close to. Please choose the incident carefully as we will be asking you further questions in the next few pages.

Please answer all the questions in your own words.

1. Please tell us about the incident or event. What happened? What was particularly difficult about the incident or event?

|  |
| --- |

1. What do you think led to or preceded the incident or event?

|  |
| --- |

1. About you:
   1. What were you doing during the incident or event?

|  |
| --- |

- 1. What was the result of your actions?

|  |
| --- |

- 1. Was this result what you wanted / had hoped for?

|  |
| --- |

- 1. Why were these actions effective or ineffective with regards to your desired outcome?

|  |
| --- |

1. About the others who were involved:
   1. What were the other people doing during the incident or event?

|  |
| --- |

- 1. What was the result or their actions?

|  |
| --- |

- 1. Do you think this result was what they were hoping for?

|  |
| --- |

- 1. Why were these actions effective or ineffective with regards to the result of their actions?

|  |
| --- |

1. What would you do if you found yourself in the same situation today?

|  |
| --- |

If discussing these incidents are making you feel upset, distressed, or anxious, please take a break. You can always close the survey and come back to it at a later date using your login details.

If you need further support please contact you care team or GP. We have also listed below a few websites that provide online support:

<https://www.beateatingdisorders.org.uk/>

<https://www.mind.org.uk/>

<https://www.rethink.org/>

<https://www.samaritans.org/>

You can also call the Samaritans crisis line free of charge if you need support:

**116 123**

You can also call the NHS 111 if you need urgent, non-emergency support.

If you feel that you are in a mental health emergency and need urgent help please go to your nearest A&E or call 999 immediately.
